# Case report: Approaches to treatment-refractory and super-refractory glutamic acid decarboxylase antibody-spectrum disorders

**DOI:** 10.3389/fimmu.2023.1297340

**Published:** 2024-01-08

**Authors:** Ravi Rajmohan, Shivali Baveja, Dai Nguyen, Eshita Shah, Michael Sy, Sanaz Attaripour, David Swope

**Affiliations:** ^1^ Department of Neurology, University of California, Irvine, CA, United States; ^2^ School of Medicine, University of California, Irvine, CA, United States; ^3^ Department of Internal Medicine, University of California, Davis, CA, United States

**Keywords:** stem cells, rituximab, stiff person syndrome, GAD-65, deep brain stimulation

## Abstract

**Background:**

Glutamic acid decarboxylase antibody-spectrum disorders (GAD-SDs) include a group of autoimmune neurological diseases associated with neuronal excitability, most noticeably stiff person syndrome. Immune modulators are the mainstay of treatment, but a significant number of patients remain refractory.

**Methods:**

We present our single-center experience of eight cases of GAD-SD, two of which were refractory to immune modulatory treatments.

**Results:**

Of the two cases that were refractory to immunomodulation, one showed significant improvement with bilateral globus pallidus interna deep brain stimulation (GPi DBS) placement, and the other showed significant improvement with autologous hematopoietic stem cell transplant (aHSCT).

**Discussion:**

To our knowledge, this is the first instance of GPi DBS placement being noted to improve GAD-SD movements.

## Introduction

Glutamic acid decarboxylase (GAD) plays a vital role in the synthesis of the inhibitory gamma-aminobutyric acid (GABA) neurotransmitters, which influence the brain’s ability to control movement, coordination, and cognition. High serum titers of autoantibodies against GAD have been associated with an array of neurological diseases (1, 2). GAD antibody-spectrum disorders (GAD-SDs) include a group of autoimmune neurological diseases associated with neuronal excitability, most noticeably stiff person syndrome (50%), cerebellar ataxia (43%), epilepsy (29%), and limbic encephalitis (16%) (1). Diagnostic criteria for simple partial seizure (SPS) include the following: “1) muscular rigidity in the limbs and axial (trunk) muscles, prominent in the abdominal and thoracolumbar paraspinals; 2) continuous co-contraction of agonist and antagonist muscles, confirmed clinically and electrophysiologically; 3) episodic spasms precipitated by unexpected noises, tactile stimuli, or emotional upset; 4) absence of any other neurologic disease that could explain stiffness and rigidity; and 5) positive anti–glutamic acid decarboxylase (GAD)65 (or amphiphysin) antibodies assessed by immunocytochemistry, Western blot, or radioimmunoassay” (3). Plasma exchange (PLEX) and intravenous immunoglobulins (IVIG) are the mainstays of disease-modifying treatment (3, 4). However, a significant number of patients are refractory to both. Below, we describe our single-center experience with refractory GAD-SD, which was observed in five of our eight patients. Two cases were deemed “super refractory”, as they did not respond to rituximab but did have partial success to bilateral globus pallidus interna deep brain stimulator placement and autologous hematopoietic stem cell transfer. Patients were asked to submit a statement from their perspective but declined to do so.

## Case presentations

Our clinic is a university-based tertiary referral center in southern California serving a population of 3 million people. Between 2018 and 2022, we identified nine individuals who were diagnosed with GAD-SD through a consensus between our neuroimmunology and movement disorder specialists based on examination findings and serum positivity and after undergoing imaging and serum studies to rule out mimics as consistent with standard-of-care guidelines (**Table 1**). Six of the seven living patients consented to the publication of their cases, and the case of one patient who declined is not discussed here. Seven of the eight patients were female, with a median age of onset of 44 ( ± 16) years and a range of 18–71. SPS was the most common presentation (75%), followed by cerebellar ataxia (25%). There was one case with epilepsy + SPS. Two of our six SPS cases had electromyography (EMG) findings consistent with diagnostic criteria (5), two had normal findings, and two others did not undergo testing. Pre-existing autoimmune disorders were seen in two cases, and underlying malignancy was suspected in two others. However, the patient with a suspected intestinal source (case 8) died from sepsis secondary to aspiration pneumonia before the diagnostic workup had been completed, and the patient with an ovarian mass had a biopsy confirmation of cystic adenoma. Five cases were refractory to IVIG and PLEX treatment, two of which had at least moderate response to rituximab. One patient who was refractory to PLEX died from sepsis secondary to chronic leukopenia in the setting of PLEX (case 6). The two cases refractory to rituximab (super-refractory cases) have been followed up for 5 years since symptom onset and roughly 2 years since receiving their “super-refractory” treatments. One had significant improvement with bilateral globus pallidus interna deep brain stimulation (GPi DBS) placement with some fluctuations, and the one that underwent autologous hematopoietic stem cell transplant (aHSCT) had total resolution of painful spasms but still had moderate impairments in function due to spasticity and dystonia. These two cases (cases 1 and 4) are described in detail in the next section.

**Table 1:** Summary of cases.

Case no.	Age of symptom onset	Sex	Pre-existing conditions	Presenting symptoms	Diagnostic confirmation	EMG consistent with SPS?	Treatments	Complications	Outcomes
1	18	F	Bx confirmed cystic adenoma ovarian mass	SPS, emotional lability	Serum GAD 2.67 nmol/L	Yes	IVIG ->PLEX ->Rituximab	Refractory status dystonicus s/p DBS placement	Significant improvement s/p DBS placement
2	34	M	History of alcohol abuse, Anxiety	SPS, hyperekplexia	Serum GAD 0.08 nmol/L	No	IVIG ->Rituximab ->IVIG	No	Moderate improvement in stiffness, mild improvement in hyperekplexia
3	37	F		SPS	Serum GAD 0.28 nmol/L	Did not have EMG	IVIG ->Rituximab	No	Stable with IVIG, awaiting further observation on rituximab
4	41	F	Epilepsy during pregnancy, Hashimoto’s thyroiditis	SPS, hyperekplexia, epilepsy	Serum GAD 136 nmol/L, gliadin 102 CU (<20.0 CU)	No	IVIG ->PLEX ->Rituximab	Refractory spasms treated with HSCT	Moderate improvement of spasms s/p HSCT
5	49	F		Cerebellar ataxia 1 month s/p flu-like illness	Serum GAD >250 IU/mL Antigliadin 23 U, (<20 U) Antithyroid abs 46.7 IU/mL (≤20 IU/mL)	N/A	PLEX ->Rituximab	no	Significant improvement
6	52	F		SPS	Serum GAD 591 nmol/L	Yes	IVIG ->PLEX	Leukopenia secondary to chronic immunosuppression	Minimal improvement before passing from leukopenia
7	53	F	RA since age 15. Primary immunodeficiency since age 49, anti-TPO, VGKC abs	SPS, OCD	Serum GAD 4.72 nmol/L	Did not have EMG	IVIG	No	Significant improvement
8	71	F		Cerebellar ataxia	Serum GAD 443 nmol/L CSF GAD 1 nmol/L	N/A	IVIG ->PLEX	G-tube placement for severe dysphagia	Minimal improvement before passing from sepsis

Reference ranges are provided in parentheses. Reference ranges for GAD-65 ab in serum and CSF are ≤0.02 nmol/L and <5 IU/mL, respectively.

Anti-TPO, thyroid peroxidase antibodies; Bx, biopsy; DBS, deep brain stimulation; GAD, glutamic acid decarboxylase; HSCT, hematopoietic stem cell transplant; IVIG, intravenous immunoglobulins; OCD, obsessive-compulsive disorder; PLEX, plasma exchange; RA, rheumatoid arthritis; s/p, “status post” (i.e., after the procedure); SPS, stiff person syndrome; VGKC abs, voltage-gated potassium channel antibodies; EMG, electromyography; CSF, cerebrospinal fluid. NA, not applicable.

### Case 1

Our first patient is a 23-year-old Asian woman who initially presented with abdominal pain, nausea, and diarrhea at age 16. A biopsy of an ovarian mass confirmed a cystadenoma. At 1.5 years after her tumor resection, the patient returned with an insidiously progressive decline in speech and writing, blepharospasm, suicidal ideation, and mood lability. Upon initial examination by a movement disorder specialist, she was noted to have dystonic movements affecting bilaterally extensor spinal musculature, knee flexors, orbicularis oculi, and lateral neck muscles.

Brain MRI was normal. Electroencephalography (EEG) was of limited value due to motion artifacts from the patient’s movements but did not identify any epileptiform abnormalities. EMG of selected muscles showed continuous firing of normally configured and recruited motor unit potentials, consistent with diagnostic criteria for SPS (3). Her serum GAD-65 ab titer returned positive at 2.67 nmol/L (reference range ≤0.02 nmol/L) with the additional finding of a singular allele for spinocerebellar ataxia type 2. Lumbar puncture revealed an opening pressure of 12 cm H_2_O, with a profile of 0 red blood cells (RBC)/mm^3^, 0 white blood cells (WBC)/mm^3^, glucose 59 mg/dL, and protein 19 mg/dL. Further extensive autoimmune/infectious cytology studies of cerebrospinal fluid (CSF) were also negative. It is important to recall that CSF samples of GAD-65 antibody titer are not considered accurate, as serum and serum measurements are the preferred standard (6). She was empirically treated with methylprednisolone and rituximab for suspected autoimmune encephalitis but showed no improvement. The patient’s symptoms were refractory to benzodiazepines, muscle relaxants, botulinum toxin, and trihexyphenidyl.

Eight months later, her serum GAD-65 ab remained at 72 IU/mL (reference range <5 IU/mL), and she was started on rituximab q6 months. Within 2 months, serum GAD-65 ab levels were undetectable (decreasing from 53 IU/mL to 6 IU/mL to <5 IU/mL), with some improvement in her hyperkinetic movements, but her symptoms returned. A repeat transvaginal ultrasound confirmed no new evidence of malignancy.

Six months afterward, she was admitted to the intensive care unit (ICU) for status dystonicus at an outside facility and had bilateral GPi DBS placement. Brain MRI at that time noted interval development of mild diffuse non-specific atrophy throughout the bilateral cerebral hemispheres and minimal atrophy of the midbrain. She had significant improvement in her large amplitude movements and could ambulate independently with a front-wheel walker and required minimal to moderate assistance for activities of daily living (ADLs).

**Figure 1:**
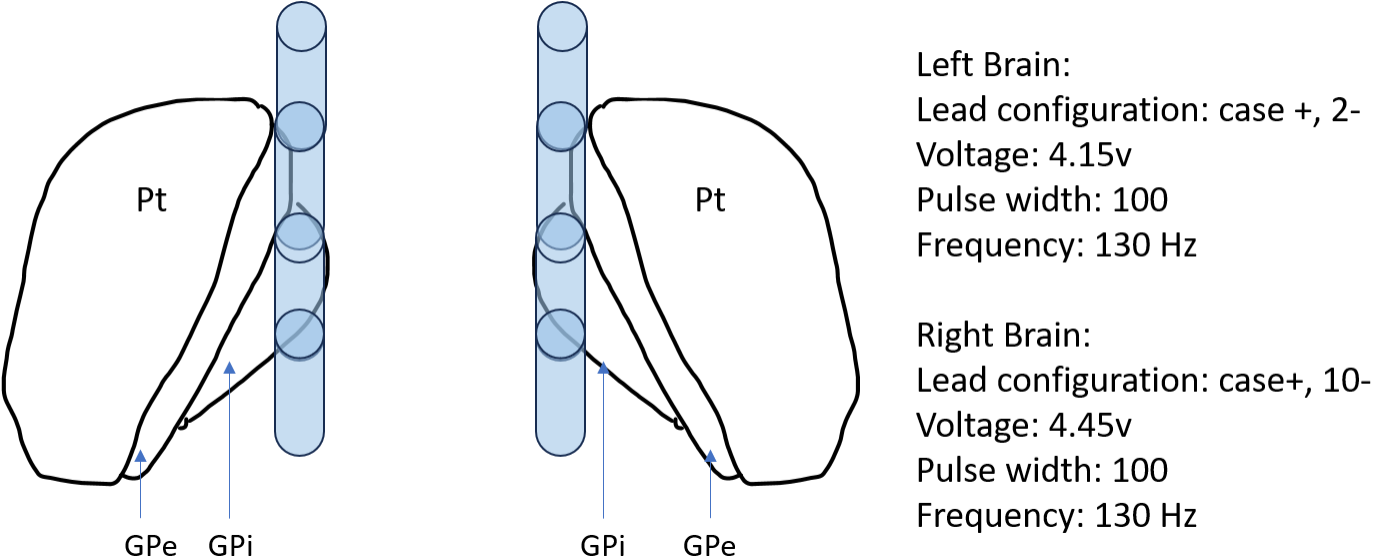
Depiction of case 1 deep brain stimulation settings. Axial depiction of Case 1 Deep Brain stimulation bilateral globus pallidus internus placement with settings. GPe, Globus pallidus externus; GPi, Globus pallidus internus; Pt, putamen; v, voltage; μs, microseconds; Hz, hertz.

Four months after discharge, she returned for acute-onset status dystonicus in the setting of DBS battery failure. She presented with rhabdomyolysis with a CK of up to 18,000 U/L (reference range 30–223 U/L). Her status dystonicus remained refractory to aggressive medical management. Her symptoms rapidly improved after battery replacement with a return to baseline within 2 days. Three months later, roughly 18 months from symptom onset, she had age-appropriate intact cognition, could speak in one- to two-word phrases, and could stand up from a chair. She underwent reprogramming of her DBS and had marked improvement in her speech, now able to speak one to two full sentences, and was able to walk 20 feet with assistance (see **Video 1**). She remains at this level of functioning, now 3 years after symptom onset. A summary of the patient’s treatment course is provided in **Table 2**. The patient's DBS settings are presented in **Figure 1**.

**Table 2:** Summary of case 1.

Case 1	April–May 2018	August–December 2018	January–April 2019			February 2021		
Serum GAD-65 ab value	2.67 nmol/L 5/2018	<5 IU/mL 8/28/2018	72 IU/mL 1/30/2019	53 IU/mL 2/8/2019	6 IU/mL 4/8/2019		0.47 nmol/L 2/17/2021
Treatments	Rituxan 1,000 mg 4/23/2018	4 doses IVIG 12/2018	Rituxan 500 mg 2/5/2019 6 doses IVIG 1/2019	Rituxan 1,000 mg 2/25/2019	Rituxan 1,000 mg 3/11/2019	DBS placed 12/16/2019	DBS battery dies, 2/25/2021
Exam findings	Blepharospasm Dysarthria	Craniofacial dystonia Stiff gait		Infrequent craniofacial movement Normal gait	Decreased frequency of hyperkinetic movements Dystonia persists	Refractory status dystonicus resolves s/p DBS	Status dystonicus resolves with battery replacement

Disease and treatment course.

DBS, deep brain stimulation; GAD-65 ab, glutamic acid decarboxylase-65 antibody; GPi, globus pallidus interna; IVIG, intravenous immunoglobulin; s/p, status post.

### Case 4

Our second patient is a 47-year-old Hispanic woman with a history of Hashimoto thyroiditis, pernicious anemia, celiac disease, and epilepsy with focal and focal to bilateral tonic–clonic seizures. A description of her case from a hematological perspective has been previously published (7). Prior to her SPS diagnosis, the patient was followed up in the epilepsy clinic after having three probable nocturnal seizures starting in the 25th week of her pregnancy in 2014. Extensive workup for an underlying etiology for her new-onset epilepsy with MRIs, EEGs, serum studies, and paraneoplastic screening was unrevealing. She remained seizure-free on levetiracetam 1,000 mg twice daily, and this was reduced to 500 mg twice daily in 2018 due to irritability. She underwent an epilepsy monitoring unit admission in 2022, which did not capture any seizures but noted that her episodes of body spasms and jerks did not have epileptiform correlates.

In 2017, SPS symptoms began with back spasms leading to multiple ground-level falls for which she sought multiple opinions and was suspected to have a functional movement disorder after EMG was within normal limits. She presented to our clinic in 2019 with stiff lumbar paraspinal muscles, left medial thigh and calf spasticity, left foot inversion, and spastic gait. Her serum GAD-65 ab was 136 nmol/L. She was diagnosed with SPS, which was refractory to IVIG and PLEX. Her condition continued to worsen despite rituximab infusions. By 2022, she underwent aHSCT with ATG/cyclophosphamide conditioning regimen and cyclophosphamide + G-CSF and aHSC collection. Her course was complicated by neutropenic fever; however, she stabilized and was discharged on day 14 of aHSCT.

By day 27 post-autologous transplant, she had complete resolution of her spasms. At 2 months of follow-up, she remained spasm-free. On the physical examination, she had residual generalized weakness and hyperreflexia. Her ambulation continued to be difficult, and repeat anti-GAD measured 40.2 nmol/L. Her condition remains stable.

## Discussion

The objective of this case series is to review potentially effective treatments for refractory and super-refractory GAD-SDs. However, there are some important differences between our cohort and prior reports that may limit the generalizability of our findings. In our cohort of patients, we saw a female:male predominance of 8:1, which is higher than most reports of roughly 6:1. Additionally, our rates of pre-existing autoimmune disorders or underlying malignancy were on the lower side of prior reports. Diagnoses were confirmed for all our patients by the presence of GAD autoantibodies, but only two of our six SPS cases had EMG confirmation. We acknowledge that, as per the description notice provided by Mayo Laboratories, “GAD65 antibody values less than 2.00 nmol/L have a lower positive predictive value for neurological autoimmunity than values of 20.0 nmol/L and higher”, and this was seen in two of our cases (cases 2 and 3) (8). However, this is often the case in patients with diabetes mellitus, thyroid disorders, or pernicious anemia (8), which neither patient had, and highlights the importance of why the diagnosis of GAD-SD is not based solely on serum antibody titers but on the combination of titers with clinical findings (3).

There was no correlation between the quantitative antibody titer and the severity of the initial presentation, consistent with previous reports and current understandings (6, 7). Seronegativity may be seen in up to 20% of cases (9), and CSF antibody testing should be considered if clinical suspicion remains high. Overall, immunomodulatory therapies were effective. Five of our cases were refractory to either IVIG or PLEX, and only two were refractory to rituximab. Unfortunately, none of the patients, regardless of their treatment, had a full return to baseline. Two patients died from sepsis but for different reasons. Case 6 developed chronic leukopenia while on PLEX, but it cannot be determined if this was a consequence of repeated PLEX therapy or due to an underlying leukemia.

Alternative fourth-line treatments were employed for two super-refractory patients. For one patient, HSCT was effective and has been previously described (4, 9–14). In our first patient, GPi DBS was placed specifically for her refractory status dystonicus in accordance with the standard-of-care treatment for refractory status dystonicus and not as an experimental approach to treating her stiff person syndrome. GPi DBS for medically refractory dystonia has been approved by the Food and Drug Administration in the United States since 2003, and two multicenter studies demonstrated clinical benefit in generalized dystonia since 2006 (15, 16). Example images of GPi DBS placement for dystonia may be found here (17). For a review of its indications and uses, see (17–20). A 2021 systematic review of medically refractory dystonia of 35 studies (n = 319) found that GPi DBS placement was consistently safe and effective and noted that primary dystonias achieved better motor symptom and disability score improvement than secondary dystonias (21). To our knowledge, this is the first reported example of GPi DBS use in GAD-SD, which suggests that it may be an effective alternative treatment for super-refractory SPS patients. Further investigation of the safety and efficacy of bilateral GPi DBS placement as a treatment for super-refractory SPS through open-label and randomized controlled clinical trials is needed to validate this possible intervention.

## Conclusion

Immunomodulatory therapies are an effective means of treatment for the majority of patients with GAD-SDs. In super-refractory cases, GPi DBS (in the context of refractory status dystonicus) and aHSCT were observed to be effective treatments. Further exploration is needed to understand their efficacy across a wider patient population of super-refractory GAD-SDs.

## Data availability statement

The original contributions presented in the study are included in the article/**Supplementary Material**. Further inquiries can be directed to the corresponding author.

## Ethics statement

Written informed consent was obtained from the individual(s) for the publication of any potentially identifiable images or data included in this article.

## Author contributions

RR: Conceptualization, Data curation, Investigation, Supervision, Writing – original draft, Writing – review & editing. SB: Data curation, Investigation, Writing – original draft. DN: Data curation, Investigation, Writing – original draft. ES: Data curation, Investigation, Writing – original draft. MS: Conceptualization, Data curation, Supervision, Writing – review & editing. SA: Conceptualization, Data curation, Supervision, Writing – review & editing. DS: Conceptualization, Supervision, Writing – review & editing.

## Supplementary material

Supplementary Video 1 Video of case 1 with deep brain stimulation (DBS) off *vs.* on. The patient presented to clinic for adjustment of her DBS settings. Note the difference in attempted ambulation when the DBS is turned off *vs.* on with the following settings. Left brain: lead configuration, c+2-; current, 4.00 mA; pulse width, 60 µs; frequency, 130 Hz. Right brain: lead configuration, c+10-; current, 4.05 mA; pulse width, 60 µs; frequency, 130 Hz.
